# Relationship between Family Variables and Career Adaptability: A Meta-Analysis

**DOI:** 10.3390/bs14090840

**Published:** 2024-09-19

**Authors:** Zhuoxi Wang, Wei Dong

**Affiliations:** 1 Moray House School of Education and Sport, University of Edinburgh, Edinburgh EH8 8AQ, UK; zhuoxiwang2000@gmail.com; 2 School of Education, Tianjin University, Tianjin 300350, China

**Keywords:** career adaptability, career development, family, parental career-related behaviors, meta-analysis

## Abstract

This study aimed to examine the relationship between family variables (i.e., family support, family SES, parental career-related behavior (PCB) support, PCB interference, and a PCB lack of engagement) and career adaptability through a meta-analysis. A systematic search for relevant studies was conducted using research databases. Twenty-four quantitative studies were yielded from 1684 records on the association between family variables and CA that were published between 1981 and 2024. Two researchers conducted data extraction independently, following coding standards. Comprehensive Meta-Analysis Version 3.3 was used in this study. The result showed that PCB support demonstrated a moderately significant correlation with CA and the largest effect size (r = 0.325). A non-significant result was found only for the correlation between PCB interference and CA. Except for gender, the moderating effects of region, age, CA measure, and publication year were found in the relationship between some family variables and CA. One limitation is the restricted range of the samples due to few studies investigating samples from regions other than Asia. The findings can highlight future directions for family studies and career research and hold practical implications for institutes, companies, and communities related to career development. This study was registered in the Open Science Framework (10.17605/OSF.IO/76HNQ).

## 1. Introduction

Career development is a lifelong process [1]. However, the unstable job market raises concerns about employability [2]. For adolescents, career preparation for school–work transition is considered one of the core developmental tasks [3]. As a self-regulation resource, career adaptability (CA) can increase the chances of finding suitable jobs during transitions [4]. When socioeconomic changes occur, individuals might experience stress in career processes [5]. Contextual factors, such as social support and opportunities, can be essential for job seekers to facilitate career planning and exploration [6]. Social support often comprises psychological and material assistance from different sources [7]. Among social networks, family can be indispensable in supporting career development [8].

### 1.1. Career Adaptability

Savickas [9] notes that adaptability is the capacity to readily adapt to novel or modified conditions. In career contexts, career adaptability is defined as “the readiness to cope with the predictable tasks of preparing for and participating in the work role and with the unpredictable adjustments prompted by changes in work and working conditions” [9] (p. 254). According to the Life-Span, Life-Space (LSLS) Theory [1], throughout their life span, individuals take on different roles (e.g., child, student, worker, and parent) in the home, the community, the school, and the workplace, showing that a career involves interacting life roles over time. Despite its broad view of life roles, the LSLS Theory is criticized for lacking the integration of different life stages [9]. Thus, Savickas [9] proposes career adaptability as a bridge across individual, developmental, self-oriented, and contextual segments in the LSLS Theory, for it explains the development from children to adults and one’s congruence with environments.

To provide a conceptual framework to elaborate on CA, Savickas [3] introduces the Career Construction Theory, highlighting that career development is driven by one’s adaptation to contexts. The theory defines four dimensions of CA: concern, control, curiosity, and confidence [3]. Specifically, career concern involves planning and preparing for future roles; career control relates to self-regulation in performing vocational tasks; career curiosity considers exploring different job options and work environments; and career confidence indicates the belief in one’s problem-solving skills during operational changes [3]. Savickas and Porfeli [10] further developed the Career Adapt-Abilities Scale (CAAS). Hou et al. [11] and Maggiori et al. [12] adapted the CAAS scale [10] and respectively designed the CAAS-China and the CAAS-Short Form (SF). Recently, numerous studies have investigated the effect of CA and proven that CA can predict career outcomes [13], for example, career aspirations [14], employability [15], and career choice satisfaction [16]. However, some research also reports that CA is not significantly associated with development in fixed working environments (e.g., salary) [17]. This controversy might suggest the role of CA as an essential resource for adapting to unfamiliar career transitions [3].

### 1.2. Family Variables

Family process variables (e.g., family support and parental behaviors) and family structural variables (e.g., family SES) can both play crucial roles in career development [18]. Research has found the positive effects of family support, parental behaviors, and family SES on CA [19,20,21].

#### 1.2.1. Family Support

With the same emphasis on the effect of environmental and individual factors on career development, the Social Cognitive Career Theory (SCCT) from Lent et al. [6] adopts Bandura’s [22] Social Cognitive Theory (SCT) and the triadic reciprocal causation model to explore the career development domain. In detail, contextual factors, individual traits, and career behaviors (e.g., CA) can mutually contribute to one another [6]. As an environmental factor, social support contains emotional, appraisal, instrumental, and informational support [7] and has been proven to facilitate CA enhancement [23]. For example, family and teachers often provide career-related information for the youth on career planning [19,24]. Peers’ psychological encouragement can also boost career confidence in decision making [25]. Research has discovered that family, teacher, and peer support have significantly positive effects on CA [26]. However, the family factor, in providing financial support in career development, might highlight its uniquely crucial role in social networks [27]. Specifically, familial instrumental support can empower adolescents to explore various educational and career activities, such as additional courses and internship opportunities [8].

#### 1.2.2. Parental Career-Related Behaviors

Family support is explained by Dietrich and Kracke [28] in an alternative way. Dietrich and Kracke [28] developed and validated an instrument for assessing perceived parental career-related behaviors (PCBs) in three dimensions, namely support, interference, and a lack of engagement, by analyzing previous qualitative research on adolescents’ reports. Self-determination Theory [29] also underscores the significance of intrinsic motivation in guiding individuals’ behaviors in life stages. Parental supportive behaviors can fulfil individuals’ autonomous needs and promote a sense of self-determination [30]. The blend of support and autonomy can help facilitate adolescents’ independence and self-regulation [31]. Parents let their offspring make career choices while offering orientation and instrumental support [32]. Parental supportive and encouraging behaviors can promote adolescents’ motivation to engage in career preparation and exploration processes [33].

By contrast, a lack of parent–child congruence on educational aspirations can cause the child’s low achievement [34]. Parents’ high aspirations for their children can lead to excessive parental involvement in educational activities [35]. Over-parenting styles often include autonomy-limiting behavioral control (e.g., academic activities) and psychological control (e.g., feelings and thoughts) [36]. Some research found that parental involvement in the career development of adolescents might be beneficial, regardless of whether the involvement was supportive or intrusive and controlling [37]. However, adolescents in over-parenting families might depend on others and exhibit maladaptive work behaviors [38]. Moreover, a lack of parental career guidance for adolescents can result in the adolescent having a less stable career path [39]. Neglectful parenting behaviors can negatively relate to adolescents’ readiness to participate in career activities [40] and their self-efficacy in career decision making [41]. Research has discovered the positive effect of PCB support [20] and the negative impacts of PCB interference [42] and a PCB lack of engagement [43] on CA.

#### 1.2.3. Family Socioeconomic Status 

Socioeconomic status (SES) comprises income, education, and occupation [8]. Family SES can be a distal antecedent of career development [44]. Parents with a higher educational attainment tend to be more prepared to actively engage in their child’s academic activities and career planning [45]. On the contrary, lower-class parents placed less emphasis on their children’s self-improvement [46]. Parents’ occupational background can also be an additional obstacle to acquiring sufficient vocational knowledge to guide their children [47]. For example, compared with parents in the working class, parents with an entrepreneurial background can offer more professional business information [48]. Apart from the indirect influence, family SES is also found to influence individuals’ CA directly [21]. Teenage career aspirations can be directly influenced by their family’s social class [49]. The planning attitude further motivates individuals’ concern for future careers [3].

### 1.3. Moderators in the Relationship between Family Variables and CA

The inconsistent results of previous studies might be due to the moderating variables affecting the relationships between family variables and CA. Sample type (region, age, and gender), CA measure, and publication year might be the moderators in the effects of family variables on CA.

Regional divergence may lead to significant differences in the relationship between family variables and CA. For example, the positive correlation between parental education and the child’s CA is significant in a study from China [50] but not in a study from Switzerland [14]. A high parental educational level in Switzerland might promote an adolescents’ life satisfaction but might not translate into adequate career preparation [14]. Moreover, family support was found to positively influence CA in a study from America [51] but not in a study from China [52]. Controlling involvement might be a typical parenting style in China [35]. The Chinese traditional parenting style might diminish the positive effect of family support on CA [52]. Therefore, the regional moderator in the relationship between family variables and CA appears to need to be examined.

Age inconsistency can potentially correlate with disagreements in the association between family variables and CA. Some research found that late adolescents seem to value parental emotional encouragement and autonomy support more highly than parental instrumental engagement [23]. In contrast, Wang et al. [53] discovered that parental support has an increasingly positive effect on CA as the youth ages. Middle and late adolescents are more likely to handle their relationships with parents with maturity compared with early adolescents, who often rebel against parental involvement [54]. Thus, the moderating effect of sample age on the correlation between family variables and CA might need to be explored.

The gender differences might result in variations in the association between family variables and CA. Gender is often regarded as a distal antecedent of career development [44]. Some findings show that the female gender might positively affect CA development over time [55]. In addition, the child’s gender also needs to be considered in research on family support [18]. For instance, Zhang et al. [56] reveal that career-related parental support exerts a stronger influence on male students than female students for career commitment and career exploration. Given the results in previous studies, whether gender is a moderator existing in the relationship between family variables and CA needs to be analyzed.

Various CA measures might lead to different effects of family variables on CA. International CA scales, such as CAAS [10] and CAAS-SF [12], were often utilized in previous studies [23,53]. Some research [57] on Chinese adolescents’ CA employed the Chinese indigenous CA measure—CAAS-China [11]. The CA studies before the development of CAAS [10] often mix different scales that are relevant to CA and develop an integrated CA measure. For instance, Kenny and Bledsoe [51] combined school identification, perceptions of educational barriers, outcome expectations, and career planning into a CA scale. The CA measure in research from Creed et al. [58] includes career concerns, career planning, career exploration, and career decision making. Therefore, the different CA measures can include different numbers of items and dimensional constructions. The diverse CA measures might moderate the results of the correlation between family variables and CA. 

Publication year can be a moderator in the relationship between family variables and CA due to recent social—contextual changes. OECD [5] suggests that the prevalence of COVID-19 in recent years poses threats to both families and individuals in multiple ways, such as causing economic strain for families, physical and mental problems, and academic and vocational stress. Parental concern and involvement in the child’s career development have increased in recent years [21]. Schools also tend to more actively develop career education programs to help adolescents establish successful career paths [59]. As the influence of family variables on CA might have changed over time, exploring the moderating effect of publication year might be imperative.

### 1.4. The Present Study

Previous empirical studies, meta-analyses, and systematic reviews concentrated on the effects of CA on career outcomes and performance [13,60]. Existing meta-analytic research also only involved the parental impact on other career competencies [61]. A meta-analysis of the association between family variables and CA is missing. Therefore, the current study aimed to meta-analytically synthesize the existing studies on the relationships between family variables, including family support, family SES, and PCB (i.e., support, interference, and the lack of engagement), and CA. Although numerous researchers have examined various family factors in CA development, the results lack consistency. Thus, the potential moderating effects of the sample type (region, age, and gender), CA measure, and publication year in the relationship between family variables and CA were considered to be investigated in this study.

## 2. Materials and Methods

This meta-analysis followed the Preferred Reporting Items for Systematic reviews and Meta-Analyses (PRISMA 2020 statement [62]) (Supplementary Material S1 and S2). The systematic review protocol was registered in the Open Science Framework (OSF) under the number identifier: DOI 10.17605/OSF.IO/76HNQ [63].

### 2.1. Sample

The data collection was processed through identification, screening, and assessment for eligibility following the PRISMA 2000 statement [62]. The literature research was conducted using the following databases: EBSCOHost, ERIC, Google Scholar, JSTOR, ProQuest, ScienceDirect, and the Web of Science. The keywords “career adaptability*” or “career adapt-ability*” or “career adapt ability” and “family*” or “parent*” or “caregiver” were combined and searched in the databases. The studies that were published from 1981 to 2024 were searched, since career adaptability was first proposed by Super and Knasel [64]. A backward search of all the studies that cited each retrieved article was conducted to locate additional studies. The final search was performed on the 10th November 2023.

### 2.2. Inclusion and Exclusion Criteria

**Figure 1:**
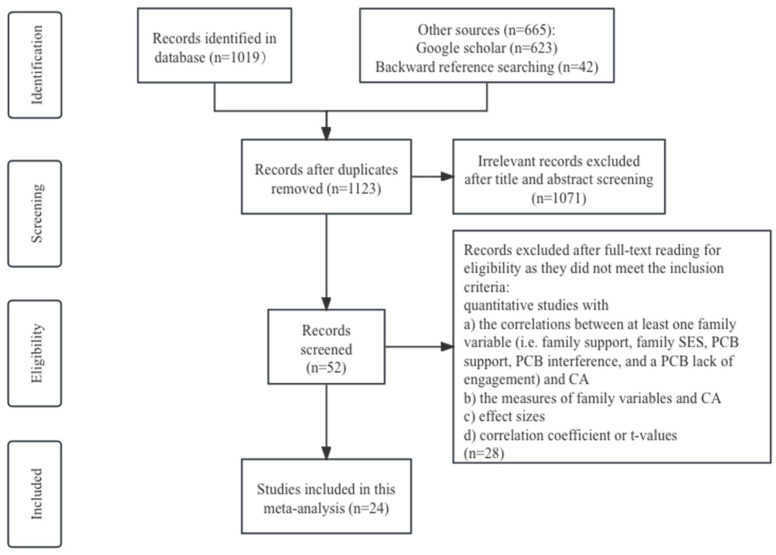
PRISMA flow chart for this meta-analysis of family variables and CA.

The included studies conformed to the following criteria: (1) It was an empirical study, including cross-sectional research and longitudinal research, providing the quantitative data related to examining the association of CA and at least one variable involving family support, family SES, and PCB. (2) The scales measuring both family variables and CA must be involved. (3) The effect sizes and correlation coefficients, or the t-values that could be converted to correlation coefficients, were presented in the study. (4) The study was published in English, in a peer-reviewed journal. Review articles, qualitative research, conference papers, and dissertations were excluded. Figure 1 shows the process of including and excluding studies. A total of 24 articles met the criteria. 

### 2.3. Data Extraction and Coding

The first author formulated preliminary coding rules as stated in this study’s purpose and according to its specific circumstances. Two researchers coded all the articles independently, following the coding standards. When discrepancies existed regarding the coding content, the two researchers reviewed the original documents and renegotiated. If a consensus could not be reached through discussion, a third independent reviewer was introduced to ensure the objectivity and transparency of the decision-making process. Records of the process and the rationale of the decisions were also documented to be tracked. The coding table was finally obtained (Table 1). The coding categories included the author, year of publication, sample size, family variables (i.e., family support, family SES, and PCB), sample regions, age stages, percentage of females, CA measures, and the correlation coefficient of the family variables on CA. The two researchers coded all the studies independently, and the initial coding consistency was 93%. 

### 2.4. Data Analysis

This study used Comprehensive Meta-Analysis Version 3.3 (CMA3.3) for the meta-analysis [74]. The correlation coefficient r as the effect size was utilized to explore the pairwise relationship between the family variables and CA. In general, 0 < r < 0.09 was considered to be nearly no effect size, 0.1 < r < 0.29 was regarded as a small effect size, 0.30 < r < 0.49 was viewed as a moderate effect size, and 0.5 < r < 1 was considered a large effect size [75]. Fisher’s Z transformation was applied to r, and the weights and 95% confidence intervals were calculated based on the sample size. Conversion formula: Zr = 0.5 × n[(1 + r)/(1 − r)], VZ = 1/n − 3, SEz = sqrt(1/n − 3), where Zr represents the converted value of the corresponding r, VZ is the variance, and SEz is the standard error. The heterogeneity analysis was evaluated using Q and I2 indicators. When Q was significant, and I^2^ was over 75%, it showed a high heterogeneity among studies [76]. A publication bias was a risk to the validity of the meta-analysis [77]. Consequently, this paper used a funnel plot, Classic Fail-safe N, and Egger’s linear regression to test for publication bias.

## 3. Results

### 3.1. Effect Size and the Homogeneity Test

Table 2 shows the results of the heterogeneity test (*Q* = 1669.143, *p* < 0.001, *I^2^* = 97.544). The data in 42 independent samples were heterogeneous, thereby confirming the appropriateness of using a random-effects model in the meta-analysis [78]. In the random effect model, the correlation between the family variables and CA was 0.128 (95%CI: 0.070~0.196, *p* < 0.001), supporting the effect of the family variables on CA.

In Table 3, there is a high heterogeneity among the research results with CA and most of the family variables: family support (*Q* = 42.090, *p* < 0.001, *I*^2^ = 80.993), family SES (*Q* = 138.948, *p* < 0.001, *I*^2^ = 92.083), PCB support (*Q* = 168.356, *p* < 0.001, *I*^2^ = 95.842), and a PCB lack of engagement (*Q* = 108.487, *p* < 0.001, *I*^2^ = 94.469). Only the studies on PCB interference did not find heterogeneity. The high heterogeneity may be due to the use of different measurement tools, sources of subjects, and various sample characteristics in the literature. Considering the heterogeneity of most of the family variables on CA, the moderators in terms of the sample types (region, age, and gender), CA measures, and publication years were examined.

**Figure 2:**
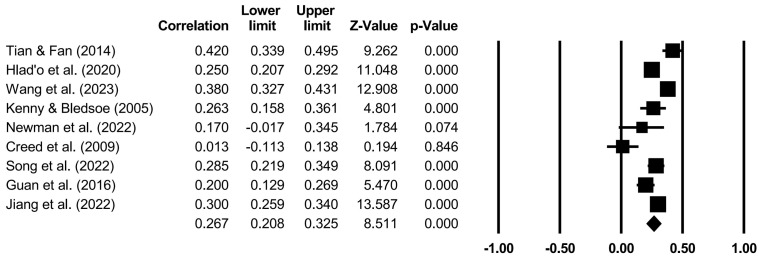
Forest plot (family support) [23,24,26,51,52,58,65,68,73].

**Figure 3:**
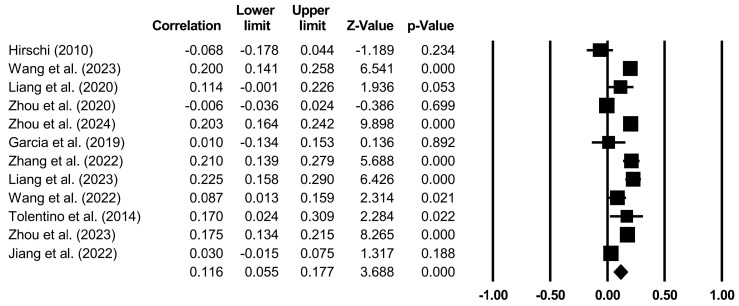
Forest plot (family SES) [14,20,26,37,42,47,59,69,70,71,72,73].

**Figure 4:**
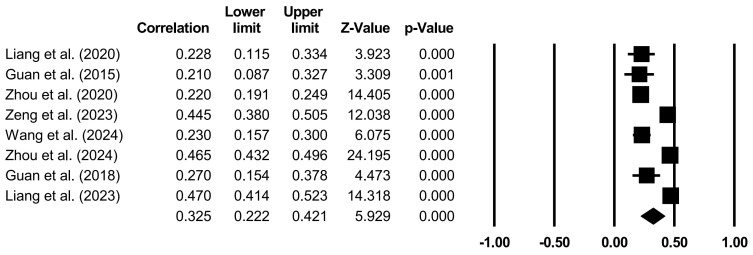
Forest plot (PCB support) [20,37,42,43,53,57,67,70].

**Figure 5:**
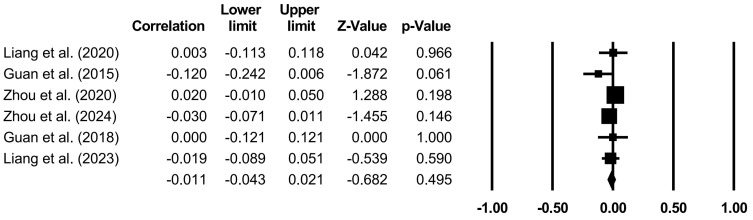
Forest plot (PCB interference) [20,37,42,43,57,70].

**Figure 6:**
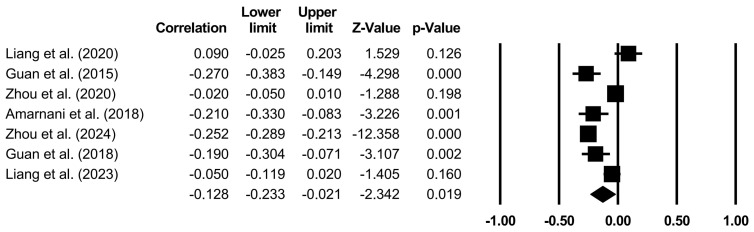
Forest plot (PCB lack of engagement) [20,37,42,43,57,66,70].

The effects of specific family variables on CA were analyzed through random models (Table 4 and Figure 2, Figure 3, Figure 4, Figure 5 and Figure 6). Family support (*r* = 0.267, *p* < 0.001), family SES (*r* = 0.116, *p* < 0.001), PCB support (*r* = 0.325, *p* < 0.001), and a PCB lack of engagement (*r* = −0.128, *p* < 0.05) were significantly correlated with CA. PCB interference (*r* = −0.011, *p* > 0.05) was not significantly correlated with CA. Among the family variables, PCB support had a moderately positive effect on CA. Family support and family SES had weakly positive effect on CA. A PCB lack of engagement had a weakly negative effect on CA. Considering the heterogeneity and the effects of most of the family variables on CA, the moderators in terms of the sample types (region, age, and gender), CA measures, and publication years were examined.

### 3.2. Publication Bias

**Figure 7:**
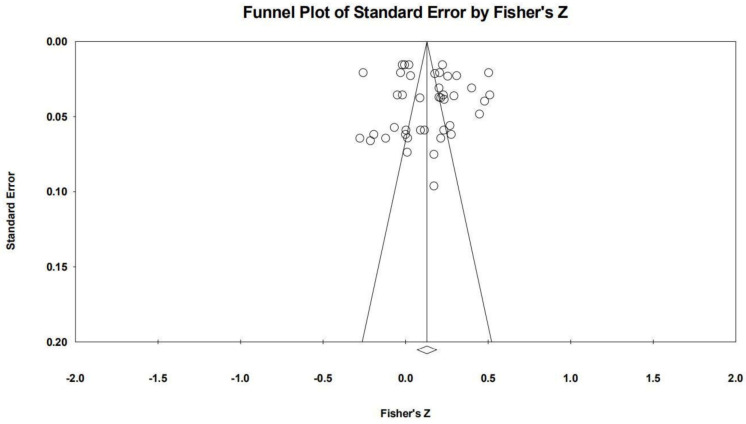
Funnel plot of the effect sizes of the correlation between the family variables and CA.

To examine whether the results were biased due to the effect sizes from various sources, a funnel plot was drawn. In Figure 7, the 42 effect sizes are symmetrically distributed on both sides of the average effect size. Since the funnel plot is an intuitive and preliminary test for publication bias, Classic Fail-safe N and Egger’s were further used for more precise results. Table 5 shows that the Classic Fail-safe N of the family variables and CA is 7168. An additional 7168 research papers were needed to overturn the results of this analysis. The *p*-values in the Egger’s test were 0.29575 and 0.59149, which are greater than 0.05, indicating no evidence of asymmetry in the funnel plot. 

### 3.3. Moderator Analysis

Random effects models were also used in the moderating effects analysis. A meta-ANOVA analysis was employed to analyze the moderating effects of the categorical variables, including sample regions, age stages, and CA measures. In contrast, the moderating effect of the continuous variables, such as the proportion of females in samples and the publication year, was tested through a meta-regression analysis.

**Figure 8:**
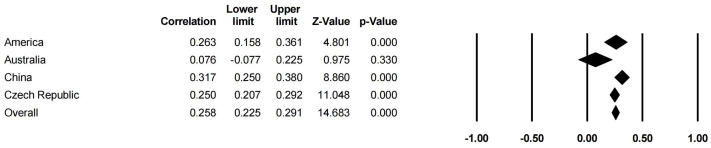
Forest plot of the region moderator (family support).

**Figure 9:**
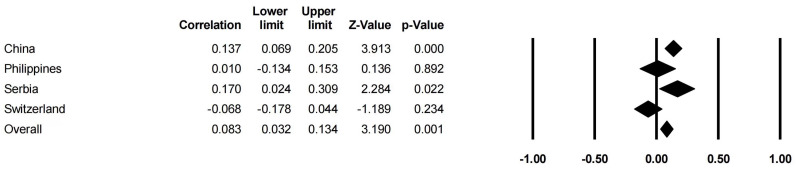
Forest plot of the region moderator (family SES).

In Table 6 and Figure 8 and Figure 9, the results show that the sample region had a stronger moderating effect on the relationships between family SES (*Q* = 11.784, df = 3, *p* < 0.05) and CA than that on the relationships between family support (*Q* = 8.958, df = 3, *p* < 0.05) and CA. However, the sample region was not the moderator of the correlation between a PCB lack of engagement (*Q* = 1.170, df = 1, *p* < 0.05) and CA. As the samples in the studies on PCB support and CA were all from China, the moderating effect of the sample region was not presented.

**Figure 10:**
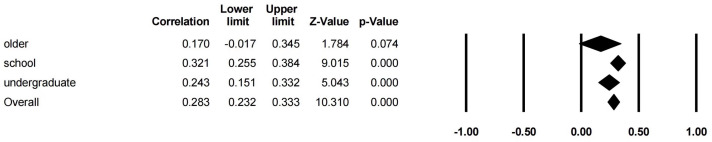
Forest plot of the age stage moderator (family support).

Table 7 and Figure 10 indicate that the age stage was only the moderator of the relationships between family support (*Q* = 3.572, df = 2, *p* < 0.05) and CA, but does not moderate the effects of family SES (*Q* = 0.434, df = 1, *p* > 0.05), PCB support (*Q* = 3.348, df = 1, *p* > 0.05), and a PCB lack of engagement (*Q* = 3.844, df = 1, *p* > 0.05) on CA.

**Figure 11:**
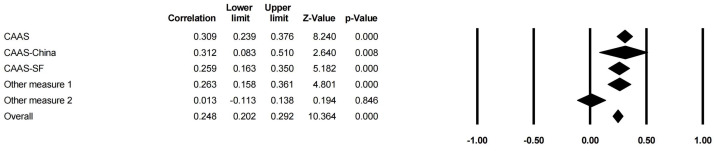
Forest plot of the CA measure moderator (family support).

**Figure 12:**
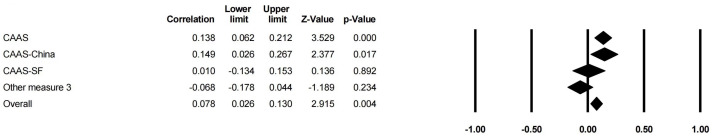
Forest plot of the CA measure moderator (family SES).

Table 8 and Figure 11 and Figure 12 explain that the CA measure had a stronger moderating effect on the correlations between family support (*Q* = 17.387, df = 4, *p* < 0.01) and CA than that on the correlations between family SES (*Q* = 11.042, df = 3, *p* < 0.05) and CA. The effects of PCB support (*Q* = 3.390, df = 2, *p* > 0.05) and a PCB lack of engagement (*Q* = 3.182, df = 1, *p* > 0.05) on CA were not moderated by the CA measure.

The meta-regression analysis demonstrated that the relationships between all the family variables, including family support (*Q_Mold_* [1, *k* = 9] = 1.16, *p* > 0.05), family SES (*Q_Mold_* [1, *k* = 12] = 0.28, *p* > 0.05), PCB support (*Q_Mold_* [1, *k* = 8] = 0.33, *p* > 0.05), and a PCB lack of engagement (*Q_Mold_* [1, *k* = 7] = 0.28, *p* > 0.05), and CA were not moderated by gender (Table 9).

**Figure 13:**
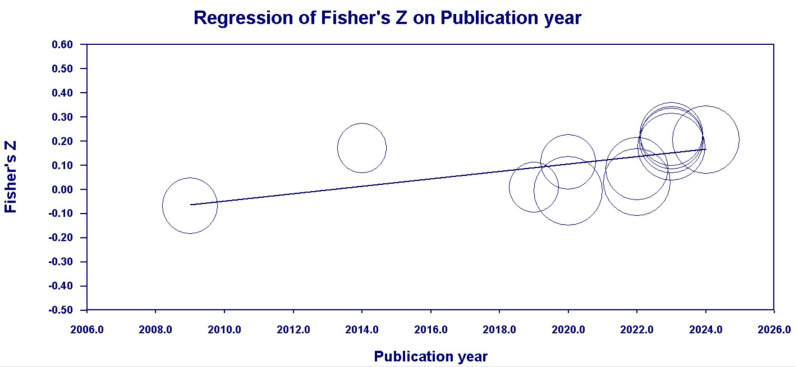
Scatter plot of the publication year moderator (family SES).

**Figure 14:**
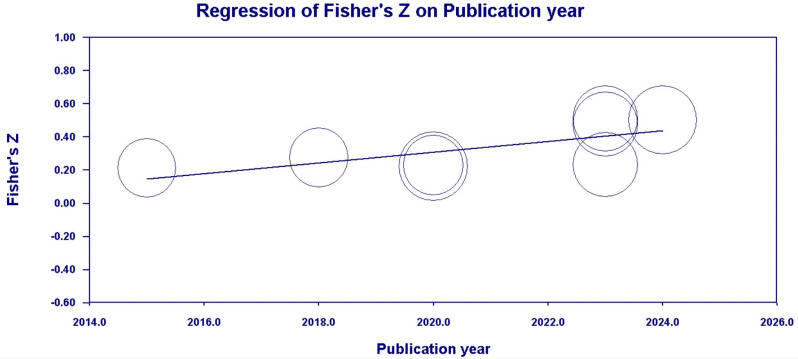
Scatter plot of the publication year moderator (PCB support).

Table 10 and Figure 13 and Figure 14 show that the publication year had a moderating effect on the correlations between family SES (*Q_Mold_* [1, *k* = 12] = 5.87, *p* < 0.05) and CA, and PCB support (*Q_Mold_* [1, *k* = 8] = 5.44, *p* < 0.05) and CA, but not on the correlations between family support (*Q_Mold_* [1, *k* = 9] = 1.71, *p* > 0.05) and CA, and a PCB lack of engagement (*Q_Mold_* [1, *k* = 7] = 0.31, *p* > 0.05) and CA.

## 4. Discussion

This meta-analysis examined previous findings on the relationships between family variables and youth’s CA. The results indicated that the four family factors (i.e., family support, family SES, PCB support, and a PCB lack of engagement) were significantly associated with CA. Still, PCB interference was not significantly correlated to CA. PCB support demonstrates a moderate association with CA, while the other three variables demonstrate a weak association with CA. The findings are in line with existing research, suggesting the effects of family support [26], family SES [42], PCB support [37], and a PCB lack of engagement [43] on CA, and support the Social Cognitive Career Theory (SCCT) that individuals’ career action can be shaped by contextual factors, such as social support and career opportunities [6].

### 4.1. The Relationship between Family Variables and Career Adaptability

#### 4.1.1. Family Support and PCB Support

As an environmental variable, family support might comprise emotional, informational, and instrumental assistance, promoting youth’s career exploration and planning [24]. Although the scales of PCB support and family support had similar components in the involved studies, the effect size of the relationships between PCB support and CA was the largest. PCB support focuses on specific career-related conduct and indicates more motivating parental career-related guidance, which is differentiated from PCB interference [28]. However, as a general concept, the family support scale might contribute to the youth’s comprehensive development and neglect the distinction between parental autonomy support and over-control in career development [24]. Research has reflected that adolescents often attach great importance to the career autonomy that their parents can provide [30]. Emotional support and encouragement can increase the youth’s willingness and confidence in career activities [43].

#### 4.1.2. Family SES

In terms of family SES, this study revealed the positive influence of family SES on CA. Career resources, information, and opportunities can be more easily accessible for adolescents with higher family SES [8]. For example, parents with entrepreneurial backgrounds can also serve as role models to stimulate the adolescents’ CA development [47]. The youth can gain vocational knowledge from the interactions between the entrepreneurs in their parents’ social networks [48]. Nevertheless, family SES had a smaller impact on CA than family support. Adolescents might not highly value the role of parental career-related instrumental actions in their CA development [23]. Moreover, parents with high external compensation values (e.g., monetary rewards, prestige, and social status) might spend more time and energy to attain extrinsic rewards from their careers, which may reduce their commitment to family roles and make them disengaged from their children’s career development [57].

#### 4.1.3. A PCB Lack of Engagement

As another constituent of PCBS, a PCB lack of engagement can inhibit adolescents’ CA [43]. A neglectful parenting style negatively affects adolescents’ readiness to participate in career activities [40] and hinders CA [3]. However, the results of this meta-analysis also revealed that a parental lack of engagement in the youth’s career might have small effects on their CA development. It might suggest that, apart from family support, peer or teacher support can also serve the function of promoting CA [26]. Specifically, some research has also found that teacher and peer support contribute more significantly to career curiosity than parental support [23]. Adolescents might often receive career-related information and positive feedback from their teachers [24] and secure attachment and encouragement on career exploration from their peers [25]. 

#### 4.1.4. PCB Interference

In contrast, the results of this study presented that the correlation between PCB interference and CA was not significant and was following the existing findings [20]. Some adolescents can still report high levels of CA and lower levels of career ambivalence when their parents are controlling and intrusive in their career preparation [37]. PCB interference appears to be conducive to the youth acquiring sufficient academic and vocational information [57]. In opposition, some research outcomes indicated the negative effect of PCB interference on CA [42]. The different discoveries might demonstrate that the impact of PCB interference on CA is controversial. First, the definitions of PCB interference and a lack of engagement can be confusing for some participants; for example, parental interference in their children’s career preparation with the neglect of the youth’s willingness might be perceived by the participants as a PCB lack of engagement [28]. Second, the literature on PCB interference analyzed in this study was all from China. Adolescents might not perceive PCB interference as negative [37], since controlling parenting practices seem to be Chinese indigenous concepts of loving and caring [31].

### 4.2. The Moderators in the Relationship between Family Variables and Career Adaptability

In this meta-analysis, four moderators were examined. First, the sample region significantly moderated the associations between family support and CA and between family SES and CA. According to the results, the correlation between family support and CA was highest in China, followed by America and the Czech Republic, and lowest in Australia. It might suggest that a family’s material and spiritual support for the youth’s development is typical in China [31]. High parental involvement in children’s development might be a profound cultural emphasis for Chinese parents [35]. The correlation between family SES and CA was highest in Switzerland and Serbia, followed by China, and lowest in the Philippines. The effect of family SES on CA appears to be more intense in developed countries than in developing countries. The research found that in some Western countries, lower-class parents stressed fewer aspirations to encourage children’s self-improvement [46]. In contrast, Chinese parents might hold their children to high expectations. Furthermore, parental aspirations for their child can mediate the relationship between social class and adolescents’ career development [49]. 

Second, sample age stages significantly moderated the correlation between family support and CA. The highest correlation was in the school stage, followed by the undergraduate stage, and the lowest was in the elder stage. As school-aged adolescents lack vocational knowledge and working experience, they might regard parents as role models and rely on parental emotional and informational support [26]. On the contrary, undergraduate students leave their parents for a long time when entering university, thus making it probable that they ask for informational assistance from their teachers [24]. Moreover, the professional challenges that the youth face might exceed their parents’ abilities to provide adequate career support [45]. 

Third, the CA measure moderated the relationships between family support and CA and between family SES and CA. Specifically, the CAAS-China had the highest impact on both relationships. It might be linked with regional adaptation. The CAAS-China was developed by Chinese scholars to suit Chinese adolescents [11]. The researchers also employed the scale in this meta-analysis to test Chinese adolescents, so it might be more accurate to measure Chinese participants’ CA. Contrarily, many scholars used either the original CAAS [10] or the short version of CAAS [12]. Some studies were conducted earlier than the CAAS was developed, combining various measures relevant to CA into one scale. 

The last moderator was the publication year. The associations between family SES and CA and between PCB support and CA were moderated by the publication year. First, the COVID-19 epidemic reduced the youth’s opportunities to start careers and led to socioeconomic changes [5]. People of a lower SES might have been more vulnerable to mental problems and more maladaptive to social stress during COVID-19 [2]. Second, the studies involved in PCB support were all from China. In recent years, Chinese parents have become increasingly responsive to child CA, which might be ascribed to the Chinese Ministry of Education’s greater attention to career education than ever before [59].

### 4.3. Implications

This study contributes to the research on the relationship between family variables and CA. Specifically, the significant effects of family support, PCB, and family SES on CA have been evidenced in existing studies. This meta-analysis synthesized previous studies and indicated that supportive parental career-related behaviors can provide encouraging career environments and opportunities for individual CA. It supports the SCCT, which stresses the effects of positive contextual factors on career behaviors [6]. This study also separately analyzed family support and PCB and demonstrated the theoretical difference between family support and PCB support. PCB support specifically stresses career-related support and can be more related to individual CA development than family support. The results underscore the complexity of family factors and highlight the potential of integrating multiple family factors and career research into future theoretical frameworks. Moreover, this study provides a broader theoretical understanding of family factors in CA development by considering the moderating effects of sample regions and ages and publication years. The family influence on CA can differ depending on the moderators. The results suggest that future theoretical frameworks in career development account for individuals of all ages in regions with various cultural norms and development levels and follow the changing career environments in different periods.

The findings also highlighted the significance of the practices from institutes, companies, and communities. This meta-analysis revealed that family SES can significantly influence CA development. Individuals in economically underdeveloped regions might enjoy less sufficient resources for career education than those in economically developed regions might possess [21]. Institutes can build home–school communication platforms and launch additional courses and activities on career development for families with lower SESs. Furthermore, companies can also provide vocational information and training programs for families in need, contributing more assistance for families to adapt to career transitions. Compared to comprehensive family support, specific career-related support from parents can be more effective for career development. Thus, communities can also provide families with career counselling on navigating career transitions. Career counsellors can focus on matching families’ demands with suitable career guidance and help them familiarize career paths through career assessments and exploration [3].

### 4.4. Limitations and Future Directions

One limitation is the restricted range of the samples. The small number of studies included in the moderator analysis might also undermine the robustness of the conclusions. For example, the methodological heterogeneity of the CA measures in the studies might influence the generalizability of the findings. Additionally, most studies in this meta-analysis were from Asia, while some were from Europe, Oceania, and North America. For example, the literature on PCB support and PCB interference was all from China. The results from other regions and cultures were not considered. Family influences on youth’s CA in different areas, such as Africa and South America, can be involved in future research. Moreover, the effect of PCB interference on CA was not significant in this meta-analysis. The generalization of the effects of family variables, such as PCB, on CA needs further testing through research with a broader range of samples. Second, the moderator of age stages was divided into school, university, and older. However, secondary vocational schools and higher vocational colleges might offer different career curriculums from general schools and universities. Previous studies have not specifically focused on the various types of institutes. Researchers can further investigate the differences in the career development of youth from different educational backgrounds. Third, most CA measures in this study were the CAAS [10] or adapted based on the CAAS. Other CA scales with new career dimensions or elements can be considered in future studies.

## 5. Conclusions

This meta-analysis study synthesized the data from 24 studies on the relationship between family variables and CA. The results revealed that PCB support demonstrated the largest effect size and a moderate association with CA. Family support, family SES, and a PCB lack of engagement demonstrated low relationships with the CA. However, PCB interference was not significantly correlated with CA. In addition, the sample region, age stage, CA measure, and publication year were the moderators in the relationship between family variables and CA. However, the sample gender was not a moderator in relationships. This meta-analysis encourages the investigation of samples from multiple contexts and the exploration of more potential family influences in CA, further leading to more supportive practices for families in need.

## Figures and Tables

**Table 1:** Characteristics of the included studies in this meta-analysis.

Studies (Year)	*N*	Family Variables	Region	Age Stages	Female%	CA Measure	*r*
[65] (2014)	431	Family support	China	University	55.8	CAAS-China	0.42
[14] (2010)	308	Family SES	Switzerland	School	50	Other measure 1 *	−0.068
[23] (2020)	1874	Family support	The Czech Republic	University	46.5	CAAS	0.25
[26] (2023)	1044	Family support	China	School	44.56	CAAS	0.38
		Family SES					0.2
[20] (2020)	290	Family SES	China	School	46.3	CAAS	0.1138
		PCB support					0.2275
		PCB interference					0.0025
		PCB lack of engagement					0.09
[43] (2015)	244	PCB support	China	University	60	CAAS-China	0.21
		PCB interference					−0.12
		PCB lack of engagement					−0.27
[37] (2020)	4151	Family SES	China	School	51.99	CAAS	−0.006
		PCB support					0.22
		PCB interference					0.02
		PCB lack of engagement					−0.02
[51] (2005)	322	Family support	America	School	53.9	Other measure 2 *	0.2625
[66] (2018)	232	PCB lack of engagement	The Philippines	University	34	CAAS	−0.21
[67] (2023)	636	PCB support	China	School	48.7	CAAS	0.445
[53] (2024)	676	PCB support	China	University	53.84	CAAS-SF	0.23
[68] (2022)	111	Family support	Australia	Older (37.76)	33.04	CAAS-SF	0.17
[42] (2024)	2315	Family SES	China	School	52.8	CAAS	0.203
		PCB support					0.464625
		PCB interference					−0.03025
		PCB lack of engagement					−0.2515
[57] (2018)	264	PCB support	China	University	70.5	CAAS-China	0.27
		PCB interference					0
		PCB lack of engagement					−0.19
[69] (2019)	187	Family SES	The Philippines	University	44.9	CAAS-SF	0.01
[47] (2022)	715	Family SES	China	University	52	CAAS-China	0.21
[70] (2023)	791	Family SES	China	School	52.4	CAAS	0.225
		PCB support					0.47
		PCB interference					−0.0192
		PCB lack of engagement					−0.05
[59] (2022)	712	Family SES	China	University	63.2	CAAS-China	0.0867
[58] (2009)	245	Family support	Australia	University	83.7	Other measure 3 *	0.0125
[24] (2022)	765	Family support	China	University	0	CAAS-SF	0.285
[71] (2014)	180	Family SES	Serbia	University	44	CAAS	0.17
[52] (2016)	731	Family support	China	University	36.1	CAAS-China	0.2
[72] (2023)	2188	Family SES	China	School	52.8	CAAS	0.175
[73] (2022)	1930	Family support	China	School	34.2	CAAS	0.3
		Family SES					0.03

* Other measure 1 includes scales of career choice readiness, career planning, career exploration, and confidence [14]. Other measure 2 includes scales of identification with school, perceptions of educational barriers, career outcome expectations, and career planning [51]. Other measure 3 includes career concerns, career planning, career exploration, and career decision making [58].

**Table 2:** Random model of the correlation between the family variables and CA.

*k*	*N*	*r*	95%CI	Heterogeneity			Tau-Squared			Test of Null
				*Q*	*p*	*I* ^2^	*Tau* ^2^	SE	Tau	*Z*
42	47,973	0.128	[0.070, 0.196]	1669.143	0.000	97.544	0.036	0.012	0.189	4.290 ***

*** *p* < 0.001.

**Table 3:** Heterogeneity test of the correlation between specific family variables and CA.

Family Variables	*k*	*N*	Heterogeneity			Tau-Squared		
			*Q*	*p*	*I^2^*	*Tau* ^2^	SE	Tau
Family support	9	7453	42.090	0.000	80.993	0.006	0.004	0.075
Family SES	12	14,811	138.948	0.000	92.083	0.010	0.006	0.101
PCB support	8	9367	168.356	0.000	95.842	0.024	0.018	0.155
PCB interference	6	8055	7.458	0.189	32.954	0.000	0.001	0.022
PCB lack of engagement	7	8287	108.487	0.000	94.469	0.019	0.017	0.137

**Table 4:** Random model of the correlation between specific family variables and CA.

Family Variables	*k*	*N*	*r*	95%CI	*Z*
Family support	9	7453	0.267	[0.208, 0.325]	8.511 ***
Family SES	12	14,811	0.116	[0.055, 0.177]	3.688 ***
PCB support	8	9367	0.325	[0.222, 0.421]	5.929 ***
PCB interference	6	8055	−0.011	[−0.043, 0.021]	−0.682
PCB lack of engagement	7	8287	−0.128	[−0.233, −0.021]	−2.342 *

* *p* < 0.05, *** *p* < 0.001.

**Table 5:** Publication bias analysis.

Classic Fail-Safe N	Egger’s Intercept	SE	Lower Limit	Upper Limit	*p*-Value (1-Tailed)	*p*-Value (2-Tailed)
7168	1.14020	2.10750	−3.11922	5.39961	0.29575	0.59149

**Table 6:** Region moderators of the association between the family variables and CA.

Family Variables	Family Support		Family SES		PCB Lack of Engagement	
	*Q_b_* (df)	*r* [LL, UL]	*Q_b_* (df)	*r* [LL, UL]	*Q_b_* (df)	*r* [LL, UL]
Region	8.958 (3) *		11.784 (3) **		1.170 (1)	
China		0.317 [0.250, 0.380]		0.137 [0.069, 0.205]		−0.116 [−0.230, 0.001]
The Philippines		n/a		0.010 [−0.134, 0.153]		−0.210 [−0.330, −0.083]
Australia		0.076 [−0.077, 0.225]		n/a		n/a
Switzerland		n/a		0.170 [0.024, 0.309]		n/a
The Czech Republic		0.25 [0.207, 0.292]		n/a		n/a
Serbia		n/a		0.170 [0.024, 0.309]		n/a
America		0.263 [0.158, 0.361]		n/a		n/a

* *p* < 0.05, ** *p* < 0.01, n/a not available.

**Table 7:** Age stage moderators of the association between the family variables and CA.

Family Variables	Family Support		Family SES		PCB Support		PCB Lack of Engagement	
	*Q_b_* (df)	*r* [LL, UL]	*Q_b_* (df)	*r* [LL, UL]	*Q_b_* (df)	*r* [LL, UL]	*Q_b_* (df)	*r* [LL, UL]
Age stage	3.572 (2) *		0.434 (1)		3.348 (1)		3.844 (1)	
School		0.321 [0.255, 0.384]		0.047 [−0.186, 0.274]		0.372 [0.235, 0.495]		−0.064 [−0.207, 0.081]
Undergraduate		0.243 [0.151, 0.332]		0.128 [0.062, 0.193]		0.235 [0.180, 0.288]		−0.223 [−0.291, −0.153]
Older		0.170 [−0.017, 0.345]		n/a		n/a		n/a

* *p* < 0.05, n/a not available.

**Table 8:** CA measure moderators of the association between the family variables and CA.

Family Variables	Family Support		Family SES		PCB Support		PCB Lack of Engagement	
	*Q_b_* (df)	*r* [LL, UL]	*Q_b_* (df)	*r* [LL, UL]	*Q_b_* (df)	*r* [LL, UL]	*Q_b_* (df)	*r* [LL, UL]
CA Measure	17.387 (4) **		11.042 (3) *		3.390 (2)		3.182 (1)	
CAAS		0.309 [0.239, 0.376]		0.138 [0.062, 0.212]		0.372 [0.235, 0.495]		−0.091 [−0.217, 0.038]
CAAS-China		0.312 [0.083, 0.510]		0.149 [0.026, 0.267]		0.241 [0.157, 0.322]		−0.229 [−0.310, −0.144]
CAAS-SF		0.259 [0.163, 0.350]		0.010 [−0.134, 0.153]		0.230 [0.157, 0.300]		n/a
Other measure 1		0.263 [0.158, 0.361]		n/a		n/a		n/a
Other measure 2		0.013 [−0.113, 0.138]		n/a		n/a		n/a
Other measure 3		n/a		−0.068 [−0.178, 0.044]		n/a		n/a

* *p* < 0.05, ** *p* < 0.01, n/a not available.

**Table 9:** Meta-regression analysis of gender (female (%)).

Family Variables	Parameter	*r* [LL, UL]	SE	*Z*
Family support	*b*_0_	−0.1744 [−0.4914, 0.1426]	0.1617	−1.08
	*b*_1_	0.3467 [0.1979, 0.4955]	0.0759	4.57
	*Q_Mold_* (1, *k* = 9) = 1.16, *p* > 0.05			
Family SES	*b*_0_	0.2543 [−0.6852, 1.1937]	0.4793	0.53
	*b*_1_	−0.0087 [−0.4762, 0.4589]	0.2386	−0.04
	*Q_Mold_* (1, *k* = 12) = 0.28, *p* > 0.05			
PCB support	*b*_0_	−0.4851 [−2.1422, 1.1719]	0.4635	−0.57
	*b*_1_	0.6010 [−0.3075, 1.5094]	0.8454	1.30
	*Q_Mold_* (1, *k* = 8) = 0.33, *p* > 0.05			
PCB lack of engagement	*b*_0_	−0.2936 [−1.3774, 0.7902]	0.5530	−0.53
	*b*_1_	0.0253 [−0.5550, 0.6055]	0.2961	0.09
	*Q_Mold_* (1, *k* = 7) = 0.28, *p* > 0.05			

**Table 10:** Meta-regression analysis of publication year.

Family Variables	Parameter	*r* [LL, UL]	SE	*Z*
Family support	*b*_0_	0.0069 [−0.0035, 0.0173]	0.0053	1.31
	*b*_1_	−13.6661 [−34.5726, 7.2405]	10.6668	−1.28
	*Q_Mold_* (1, *k* = 9) = 1.71, *p* > 0.05			
Family SES	*b*_0_	0.0153 [0.0029, 0.0277]	0.0063	2.42
	*b*_1_	−30.8709 [−55.9305, −5.8144]	12.7857	−2.41
	*Q_Mold_* (1, *k* = 12) = 5.87, *p* < 0.05			
PCB support	*b*_0_	0.0321 [0.0051, 0.0591]	0.0138	2.33
	*b*_1_	−64.5544 [−119.0663, −10.0425]	27.8127	−2.32
	*Q_Mold_* (1, *k* = 8) = 5.44, *p* < 0.05			
PCB lack of engagement	*b*_0_	0.0114 [−0.0287, 0.0514]	0.0204	0.56
	*b*_1_	−23.1194 [−104.0234, 57.7845]	41.2783	−0.56
	*Q_Mold_* (1, *k* = 7) = 0.31, *p* > 0.05			

## Data Availability

All data and materials used have been provided in the Appendix A.

## Supplementary Materials

The following supporting information can be downloaded at: https://www.mdpi.com/article/10.3390/bs14090840/s1. Supplementary Material S1: PRISMA 2020 Abstract Checklist. Supplementary Material S2: PRISMA 2020 Checklist [79].

## Appendix A

Detailed information on the source studies included in the analysis can be found at the following link: “https://docs.google.com/spreadsheets/d/1YDcYA5bI1fhQMQvHKIEw3QhjAN0egAWcwx9C12ybxgE/edit?usp=sharing”, accessed on 16 September 2024.
